# Ameloblastic Fibro-Odontoma: A Diagnostic Challenge

**DOI:** 10.1155/2010/104630

**Published:** 2010-08-19

**Authors:** Elen de Souza Tolentino, Bruna Stuchi Centurion, Marta Cunha Lima, Patrícia Freitas-Faria, Alberto Consolaro, Eduardo Sant'ana

**Affiliations:** ^1^Bauru Dental School, University of São Paulo, Alameda Octávio Pinheiro Brisola 9-75, Bauru, São Paulo 17012-901, Brazil; ^2^Stomatology Department, Bauru Dental School, University of São Paulo, Alameda Octávio Pinheiro Brisola 9-75, Bauru, São Paulo 17012-901, Brazil

## Abstract

An 11-year-old girl presented to our department to have a second opinion regarding a lesion involving her left mandible. She had previously undergone several radiographic exams including panoramic, helical, and cone-beam computed tomography. Radiographic examinations revealed a well-defined radiolucent region, which contained an irregular radiopaque mass of 3 cm in diameter, localized to the left angle of the mandible. Our presumptive diagnosis was complex odontoma. Excisional biopsy was performed, and microscopic features showed strands and islands of odontogenic epithelium showing peripheral palisading and loosely arranged central cells, identical to stellate reticulum, embedded in a myxoid cell-rich stroma resembling the dental papilla. Dentin and enamel were also presented. The diagnosis was ameloblastic fibro-odontoma, which is a rare mixed odontogenic tumor, derived from epithelial and ectomesenchymal elements that form the dental tissues.

## 1. Introduction

Ameloblastic fibro-odontoma (AFO) is a benign, slow growing, expansile epithelial odontogenic tumor with odontogenic mesenchyme. It may inhibit tooth eruption or displace involved teeth although teeth in the affected area are vital [1–3]. Radiography shows a well-defined, radiolucent area containing various amounts of radiopaque material of irregular size and form [4, 5]. The lesions are usually diagnosed during the first and second decades of life [4–6]. It occurs with equal frequency in the maxilla and the mandible and with equal frequency in males and females [6]. 

Microscopically, the lesion is composed of strands, cords, and islands of odontogenic epithelium embedded in a cell-rich primitive ectomesenchyme, resembling the dental papilla. Many authors reported that AFO is not aggressive and can be treated adequately through a surgical curettage to the lesion without removal of the adjacent teeth [1, 4, 5, 7, 8]. This paper describes an extensive AFO in an 11-year-old girl.

## 2. Case Description

An 11-year-old girl presented to our department on referral from another dentist to have a second opinion about a lesion involving the left mandible. She had radiographic examinations, including panoramic, helical, and cone-beam computed tomography. These examinations were accompanied by a presumptive radiographic differential diagnosis of “odontoameloblastoma”: complex odontoma and AFO. 

The medical, social and family histories were unremarkable, as were the results of a review of systems and a physical examination. The clinical examination did not display any sign of pain or swelling in the left mandible. There was no history of local trauma or infection. Oral inspection revealed good oral hygiene. 

**Figure 1:**
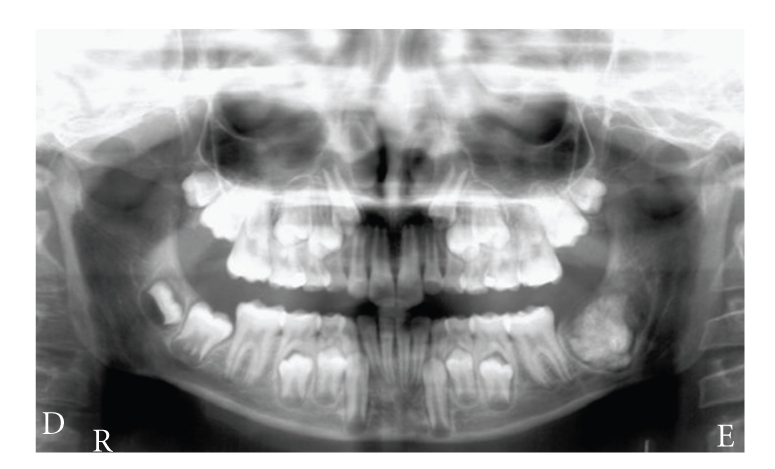
Initial panoramic radiography showing well-defined radiolucent region, which contained an irregular radiopaque mass measuring 3 cm in diameter. This lesion occupied a zone from the lower left second molar area to the left ramus.

The initial panoramic radiography revealed a well-defined radiolucent region, which contained an irregular radiopaque mass 3 cm in diameter. This lesion occupied a zone from the lower left second molar area to the left ramus. The mandibular left second molar was not present (Figure 1).

**Figure 2:**
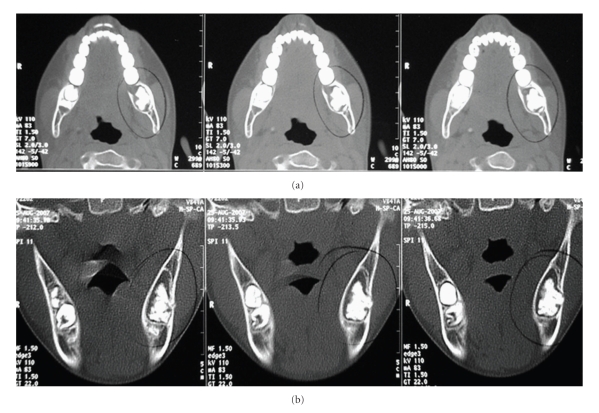
Helical computed tomography images showing an expansile, well-circumscribed lesion containing a calcified mass compatible with odontogenic tissue ((a) axial cuts; (b) coronal cuts).

**Figure 3:**
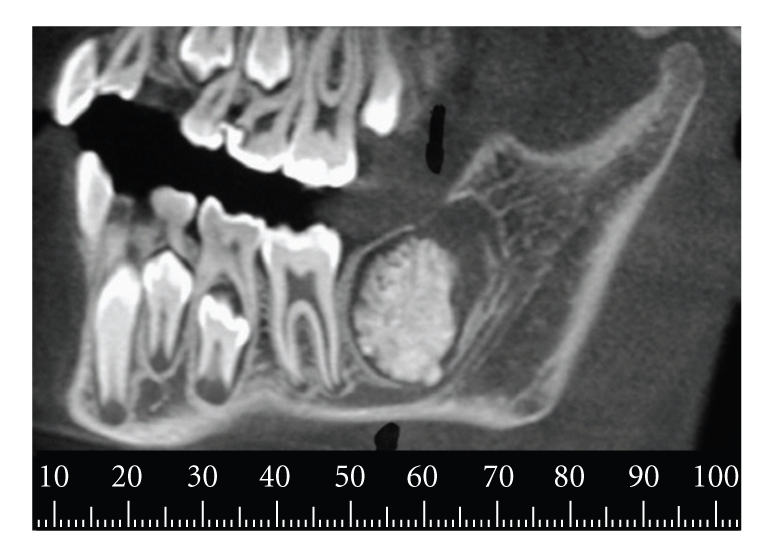
Cone-beam tomography (panoramic reconstruction) showing a well-circumscribed calcified mass in intimate contact with the alveolar inferior nerve.

Helical and cone-beam computed tomography showed an expansile well-circumscribed lesion containing at the interior a calcified mass compatible with odontogenic tissue (Figures 2 and 3). 

Considering the clinical and radiographic examinations, our presumptive diagnosis was complex odontoma.

**Figure 4:**
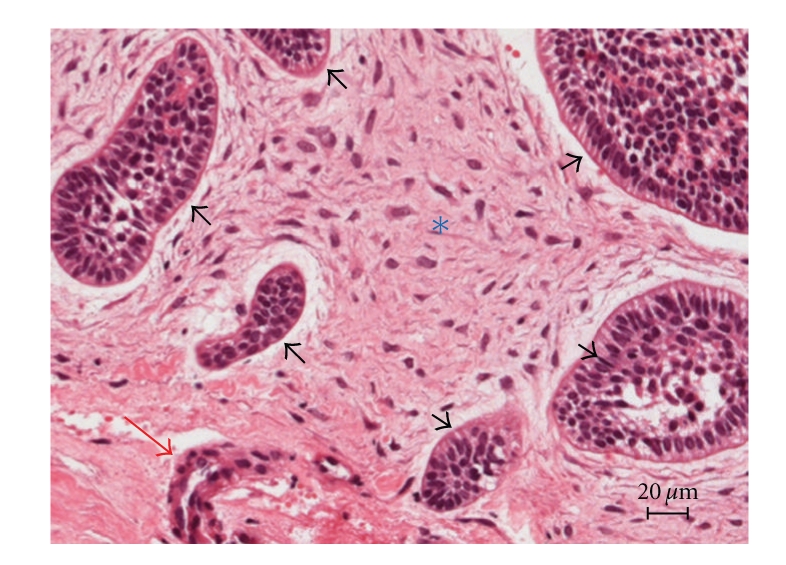
(H.E.) Strands (red arrow) and islands (black arrows) of odontogenic epithelium showing peripheral palisading and loosely arranged central cells, identical to stellate reticulum embedded in myxoid cell-rich stroma resembling the dental papilla (*).

**Figure 5:**
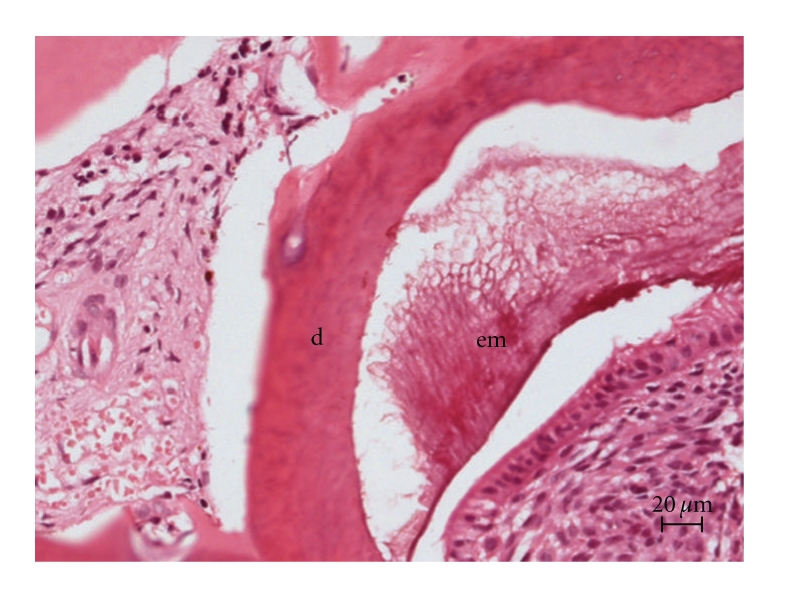
(H.E.): Dentin (d) and enamel matrix (em).

The patient underwent enucleation of the lesion and careful curettage of the surgical cavity under general anesthesia. The surgical specimen was fixed in neutral buffered 10% formalin and subjected to pathological analysis. Light microscopic examination of sections stained with hematoxylin and eosin revealed strands and islands of odontogenic epithelium showing peripheral palisading and loosely arranged central cells, identical to stellate reticulum, embedded in a myxoid cell-rich stroma resembling the dental papilla (Figure 4). Dentin and enamel were also present (Figure 5). The final diagnosis was AFO. The patient is being followed up postoperatively and there is no sign of recurrence.

## 3. Discussion

In the present case, the patient presented to our department with previous examinations, including panoramic, helical and cone-beam computed tomography. While these radiographic examintions were given a presumptive diagnosis of odontoameloblastoma by the examinaing radiologists, we believed that the findings were more common in this region. Odontoameloblastoma, also known as ameloblastic odontoma, has a more aggressive behavior, similar to an ameloblastoma rather than an odontoma [9]. 

The histogenesis of this lesion is controversial. AFO is a benign tumor that exhibits the same benign biologic behavior as that of ameloblastic fibroma, showing inductive changes that lead to the formation of both dentin and enamel [1]. This is in contrast to the ameloblastoma. Conversely, the term “odontoameloblastoma” (or “ameloblastic odontoma”) refers to tumors representing a histological combination of ameloblastoma and complex odontoma, which behave in the invasive manner of classic ameloblastoma [6].

According to the revised World Health Organization (WHO) classification [10], ameloblastic fibroma and AFO are believed to be stages of complex odontoma formation [1]. This means that the aforementioned lesions should not be considered as distinct entities [11]. Cahn and Blum [13] postulated that ameloblastic fibroma (the histologically least differentiated tumor) develops first into a moderately differentiated form, following AFO and eventually into a complex odontoma.However, the concept that these lesions represent a continuum of differentiation is not widely accepted, with other researchers suggesting that they are separate pathologic entities [12–15]. In some studies, the term AFO represents a histological combination of ameloblastic fibroma and complex odontoma [12, 16]. The majority now agrees that AFO exists as a distinct entity, but it can be histologically indistinguishable from immature complex odontoma. The arrangement of the soft tissues and the development stage of the involved tooth are useful criteria for diagnosis [3]. Despite numerous efforts, however, there is still considerable confusion concerning the nature of these lesions [17]. 

AFO is relatively rare, with the prevalence among oral biopsies being about 1% [4] and its frequency among odontogenic tumors being reported at 1% to 3% [3, 18]. This lesion usually occurs in people less than 20 years old, and age is thus an important characteristic in the differential diagnosis. This lesion is usually found in the molar area [6, 12], and the distribution is roughly equal between the maxilla and mandible [6, 12]. 

Many authors reported that AFO can be treated adequately through a surgical curettage without removal of the adjacent teeth [1, 4, 5, 7, 8]. As noted in the literature, not all lesions previously classified as AFO are, in fact, aggressive lesions. If there is a recurrence accompanied by a change of the histological pattern toward a more unorganized fibrous stroma with displacement of the epithelial component, then more extensive treatment procedures appear to be indicated [19]. Determination of a case-dependent treatment plan may provide an optimum outcome. Long-term follow up with short intervals should be maintained in the management of AFO.
